# Late endocrine-metabolic complications in survivors of young adult and adult-onset cancers: comprehensive evaluation and strategies for management. An Italian Association of Medical Oncology (AIOM), Italian Association of Medical Diabetologists (AMD), Italian Society of Diabetology (SID), Italian Society of Endocrinology (SIE), Italian Society of Pharmacology (SIF) multidisciplinary critical review

**DOI:** 10.1007/s40618-025-02764-5

**Published:** 2025-12-03

**Authors:** Francesco Felicetti, Rossella Mazzilli, Stefania Gori, Marco Gallo, Alessio Cortellini, Stella D’Oronzo, Valerio Gristina, Antonella Argentiero, Riccardo Candido, Marzia Del Re, Stefano Fogli, Tindara Franchina, Dario Giuffrida, Giampiero Marino, Matteo Monami, Monica Montagnani, Lelio Morviducci, Annalisa Natalicchio, Alberto Ragni, Valerio Renzelli, Laura Sciacca, Enzo Tuveri, Francesco Giorgino, Matteo Verzé, Maria Chiara Zatelli, Emanuela Arvat, Francesco Perrone, Fabrizio Stracci, Gianluca Aimaretti, Angelo Avogaro, Raffaella Buzzetti, Nicola Silvestris, Antongiulio Faggiano

**Affiliations:** 1 Division of Oncological Endocrinology, Department of Oncology, University Hospital A.O.U. “Città della Salute e della Scienza di Torino”, Torino, 10126 Italy; 2 Endocrinology Unit, Department of Clinical & Molecular Medicine, Sant’Andrea Hospital, Sapienza University of Rome, Rome, Italy; 3 Medical Oncology, IRCCS Sacro Cuore Don Calabria Hospital, 37024 Negrar di Valpolicella, Verona, Italy; 4 Endocrinology and Metabolic Diseases Unit, Azienda Ospedaliero- Universitaria SS Antonio e Biagio e Cesare Arrigo of Alessandria, Alessandria, Italy; 5 Operative Research Unit of Medical Oncology, Fondazione Policlinico Universitario Campus Bio-Medico, Via Alvaro del Portillo 200, Roma, 00128 Italy; 6 Department of Medicine and Surgery, Università Campus Bio-Medico di Roma, Roma, Italy; 7 Department of Surgery and Cancer, Hammersmith Hospital Campus, Imperial College London, London, UK; 8 Medicine and Surgery Department, LUM University, Casamassima, Bari, Italy; 9 Oncology and Oncohematology Division F. Miulli General Regional Hospital, Acquaviva delle Fonti, Bari, Italy; 10 Medical Oncology Unit, Department of Precision Medicine in Medical, Surgical and Critical Care, AOUP Paolo Giaccone University Hospital, University of Palermo, Palermo, Italy; 11 Medical Oncology Department, IRCCS Istituto Tumori “Giovanni Paolo II”, Bari, Italy; 12 Department of Medical Surgical and Health Sciences, University of Trieste, Trieste, 34149 Italy; 13 Saint Camillus International University of Health and Medical Sciences, Rome, Italy; 14 Direzione Scientifica, Policlinico Agostino Gemelli IRCCS, Rome, Italy; 15 Clinical Pharmacology and Pharmacogenetics Unit, Department of Clinical and Experimental Medicine, University of Pisa, Pisa, Italy; 16 Medical Oncology Unit, Department of Human Pathology “G. Barresi”, University of Messina, Messina, Italy; 17 Department of Oncology, Istituto Oncologico del Mediterraneo, Viagrande, Catania Italy; 18 Internal Medicine Department, Ospedale dei Castelli, Asl Roma 6, Ariccia, RM Italy; 19 Diabetology, Careggi Hospital and University of Florence, Florence, Italy; 20 Department of Precision and Regenerative Medicine and Ionian Area, Section of Pharmacology, University of Bari Aldo Moro, Bari, Italy; 21 Diabetology and Nutrition Unit, Department of Medical Specialties, ASL Roma 1 – S. Spirito Hospital, Rome, Italy; 22 Department of Precision and Regenerative Medicine and Ionian Area, Section of Internal Medicine, Endocrinology, Andrology and Metabolic Diseases, University of Bari Aldo Moro, Bari, Italy; 23 Diabetologist and Endocrinologist, Italian Association of Clinical Diabetologists, Rome, Italy; 24 Department of Clinical and Experimental Medicine, Endocrinology Section, University of Catania, Catania, Italy; 25 Diabetology, Endocrinology and Metabolic Diseases Unit, ASL-Sulcis, Iglesias, Cagliari, Italy; 26 Medical Direction, IRCCS Sacro Cuore Don Calabria Hospital, Negrar di Valpolicella, Verona, 37024 Italy; 27 Section of Endocrinology, Geriatrics and Internal Medicine, Department of Medical Sciences, University of Ferrara, Ferrara, Italy; 28 Clinical Trial Unit, National Cancer Institute IRCCS Fondazione Pascale, Napoli, Italy; 29 Department of Medicine and Surgery, University of Perugia, Perugia, Italy; 30 Endocrinology, Department of Translational Medicine, Università del Piemonte Orientale, Novara, Italy; 31 Fondazione Diabete Ricerca SID, Rome, Italy; 32 Diabetology, Department of Experimental Medicine, Sapienza University of Rome, Rome, Italy

**Keywords:** Breast cancer, Prostate cancer, Lung cancer, Colorectal cancer, Survivors, Adult-onset cancer

## Abstract

**Background:**

Cancer survivors have been considered individuals who have completed anti-tumor treatment and are in “remission”. However, the definition is increasingly seen as insufficient due to a significant number of patients living with chronic or stable disease, as a result of advanced therapies, and according to a recent definition, “survivors” are all people living with and beyond cancer. Breast, prostate, lung and colorectal cancers are the most frequent tumors diagnosed in Europe with an increasing population of survivors. The longer life expectancy has made it necessary to assess the health status, comorbidities, and complications in cancer patients, mainly in the age range of 20–50 years. In particular, the long-lasting hormonal therapies in hormone-sensitive tumors and the immunotherapies, that are changing the cancer clinical scenario, have opened a broad landscape of late endocrine/metabolic toxicities.

**Purpose:**

The aim of the present manuscript is to evaluate the late endocrine-metabolic complications in survivors of young adult or adult-onset cancers in the most prevalent tumors, analyzing risk factors for endocrine/metabolic disease, attempting to provide a indications for long-term surveillance and treatment strategies.

**Conclusions:**

This paper highlights the importance of recognizing endocrine and metabolic complications, as well as identifying key risk factors that can suggest a more effective surveillance and management.

## Introduction

In 2022, 4,141,360 new cancers (excluding non-melanoma skin cancer) were diagnosed in Europe, 397,618 of whom in people aged 20–49 years. Overall, breast, prostate, lung and colorectal cancers are the most frequent tumors diagnosed in Europe (Table 1).

Cancers diagnosed in people aged 20–49 years represent 9.6% of new tumors in Europe (Table 2). Incidence and even more prevalence are estimated to be increasing worldwide and an increasing number of cases has been reported also for early-onset cancers (< 50 years) [1]. Over the past three decades, the number of new cancer cases in patients < 50 years has increased by 79% globally [2]. Early-onset breast, tracheal, bronchus and lung, stomach and colorectal cancer (CRC) showed the highest mortality and burden in 2019. Countries with a high-medium and medium Sociodemographic Index and individuals aged 40–49 years were particularly affected and dietary risk factors (diet high in red meat, low in fruit, high in sodium and low in milk, etc.), alcohol consumption and tobacco use are the main risk factors underlying early-onset cancers. In comparison to later-onset cancer, the increase of cancer in adults < 50 years also has more significant effects on the social level, in addition to the personal level.

**Table 1:** Breast, prostate, lung and colorectal cancers: incidence in Europe in 2022, according to age at diagnosis (all age and subgroup age 20–49 years)

	Europe	
	All age	Age 20–49 years
Breast cancer	546,618/4,141,360 (13.2%)	105 331/397,618 (26.5%)
Prostate cancer	144,215/4,141,360 (3.5%)	2245/397,618 (0.6%)
Lung cancer	475,918/4,141,360 (11.5%)	15 820/397,618 (4.0%)
Colorectal cancer	530,635/4,141,360 (12.8%)	24,964/397,618 (6.3%)

Source: Cancer Today/IARC, Globocan 2022 (version 1.1) – 08/02/2024 https://gco.iarc.who.int

**Table 2:** Cancer incidence in Europe in 2022, in all age and in age 20–49 years (% of all ages incident cases)

All cancers, excluding non-melanoma skin cancer		
Sex	All age (0–85 + years)	20–49 years
Both	4,141,360	397,618 (9.6%)
Males	2,167,231	140,533 (6.5%)
Females	1,974,129	257,085 (13.0%)

Source: Cancer Today/IARC, Globocan 2022 (version 1.1) – 08/02/2024 https://gco.iarc.who.int

According to a recent definition, cancer survivors are all the people living with and beyond cancer. Indeed, a person is considered a cancer survivor from the time of diagnosis through the lifespan. There are many types of survivors, including those living with cancer and those free of cancer (https://www.cancer.gov/about-cancer/coping/survivorship). Due to the increased survival rates in most cancer diagnoses, cancer survivors represent a growing population, with specific and different health needs depending on prognosis, the treatments received and time since diagnosis. Systematic estimates of cancer prevalence by country are provided by IARC only for short-term follow up within 5 years from diagnosis. Complete prevalence, including all people living longer than 5 years after a cancer diagnosis, is not routinely available in Europe. Recently, complete cancer prevalence in Europe in 2020 was reported [3] using the information of the EUROCARE-6 database on patients diagnosed with cancer between 1978 and 2015 and followed until December 31, 2016, at the latest. The EUROCARE-6 dataset covers 64% of the population of the 29 participating countries. In 2020, 23 711 000 people (5% of the population) were estimated to be alive after a cancer diagnosis in Europe (12 818 000 female and 10 892 000 male). The top five tumors in female survivors were breast cancer, CRC, uterine cancer, skin melanoma, and thyroid cancer. Prostate cancer, CRC, urinary bladder cancer, skin melanoma, and kidney cancer were the most common tumors in male survivors. The differences in prevalence between countries were large and in line with the demographic structure, incidence, and survival patterns. Between 2010 and 2020, the number of prevalent cases increased by 3.5% per year (41% overall), partly due to an ageing population. Authors did not report complete prevalence data in the age class 20–49 years.

In Europe, all 5-year survivors in 2022 were 12,493,752: 1,589,451 were 20–49 years old at diagnosis (12.7%) (Table 3). The 5-year prevalence in 2022 in Europe for breast, prostate, lung and CRC, according to age at diagnosis is reported in Table 4.

**Table 3:** Cancer prevalence at 5 year in Europe in 2022, in all age and in age 20–49 years (% of all ages prevalent cases)

All cancers, excluding non-melanoma skin cancer		
Sex	All age (0–85 + years)	20–49 years
Both	12,493,752	1,589,451 (12.7%)
Males	6,224,690	529,593 (8.5%)
Females	6,269,062	1.056,858 (16.9%)

Source: Cancer Today/IARC, Globocan 2022 (version 1.1) – 08/02/2024 https://gco.iarc.who.int

**Table 4:** Breast, prostate, lung and colorectal cancers: prevalence at 5 years in Europe according to age at diagnosis

	Europe	
	Age at diagnosis of cancer	
*N* (%)	All age	Age 20–49 years
Breast cancer	2,296,495/12,493,752 (18.4%)	461,524/1,589,451 (29%)
Prostate cancer	1,915,449/12,493,752 (15.3%)	10,113/1,589,451 (0.6%)
Lung cancer	610,169/12,493,752 (4.9%)	29,862/1,589,451 (1.8%)
Colorectal cancer	1,698,014/12,493,752 (13.6%)	89,242/1,589,451 (5.6%)

Source: Cancer Today/IARC, Globocan 2022 (version 1.1) – 08/02/2024 https://gco.iarc.who.int

Considering these prevalence data and the increased survival rates following diagnosis (especially for younger patients at cancer diagnosis), it is becoming progressively more critical to manage the toxicities associated with oncological treatments, as well as any comorbidities, which may have a negative impact on the long-term survival of these individuals [4, 5].

In this paper, a panel of experts from the Italian Association of Medical Oncology (AIOM), Italian Association of Medical Diabetologists (AMD), Italian Society of Diabetology (SID), Italian Society of Endocrinology (SIE), and Italian Society of Pharmacology (SIF) provides a critical multidisciplinary review of the late endocrine-metabolic complications in survivors of young adult and adult-onset breast, prostate, lung and CRC cancers, analyzing risk factors with the aim of providing suggestions for the long-term surveillance and clinical management of these patients.

## Methods

A narrative review was conducted due to the scarcity of methodologically robust guidelines on the topic. A comprehensive search of PubMed and EMBASE was undertaken to identify current guidelines, systematic reviews, and meta-analyses using the main relevant keywords. Eligible documents included publications providing evidence-based recommendations or expert consensus on the clinical questions addressed. When clear and concordant recommendations were issued by the Societies represented within the working group, these were directly reported in the manuscript. In the absence of such recommendations, or in cases of conflicting guidance, suggestions were developed through a pragmatic approach based on discussion and consensus among the members of the working group.

### Breast cancer survivors

#### Epidemiology and definition of disease-specific breast cancer survivors

Breast cancer is the most common solid malignancy in females worldwide. In 2020, over 2.3 million new cases and 685,000 breast cancer-related deaths were reported, but the incidence of this tumor is projected to grow by over 40% in the next 15 years, reaching about 3 million cases every year, due to both population growth and ageing.

Estimates of the new cases of invasive breast cancer in the US in 2024 reported approximately 310,720 new diagnoses and 42,250 deaths, mostly occurring in women aged 50 years and older [6]; however, statistics of this malignancy in the younger population (20–49 years) are not irrelevant (with a number of deaths of 140,952 in this age range (https://gco.iarc.who.int/today/en/dataviz/barsmode=cancer&key=total&group_populations=1&types=1&sexes=0&sort_by=value0&populations=900&multiple_populations=0&values_position=out&cancers_h=15&age_start=4&age_end=9), as shown in Tables 1 and 4, which describe the current situation in European countries.

Due to the high prevalence of this disease, brought about by earlier diagnosis and more effective therapies, survivorship has become a critical issue to be addressed. The American Cancer Society reports that, currently, there are about 4 million breast cancer survivors only in the United States [7].

Breast cancer survivorship is described as including those patients “living with or through or beyond a breast cancer diagnosis” [8]. For clinical purposes, cancer survivors are often defined as patients who have been cancer-free for at least 5 years after treatment completion. In some studies, the expression is used to indicate anyone who has completed primary breast cancer therapy, except endocrine therapy, and is still alive [9].

Up to 30% of breast cancer survivors may experience both treatment-related side effects and cancer-related symptoms, which can negatively affect quality of life (QoL) [8].

For this reason, the American Cancer Society/American Society of Clinical Oncology (ACS/ASCO) Breast Cancer Survivorship Care Guideline provides recommendations to manage adult survivors of breast cancer, suggesting long-term monitoring of potential physical and psychosocial effects of breast cancer and its treatment [10].

#### Risk factors for endocrine/metabolic disease in breast cancer survivors

Several potential endocrine late complications from breast cancer have been described, including the onset of diabetes mellitus (DM) [11, 12], thyroid and parathyroid disorders [13], as well as bone loss and menopause-related symptoms [14, 15]. Patients with oestrogen receptor expressing breast cancers represent the category at highest risk of endocrine and metabolic complications. Firstly, aromatase inhibitors, often following a chemotherapy-induced menopause, together with GnRH agonists, are the main cause of a significant bone loss and an increased risk of fragility fractures, largely independent from bone mineral density [16]. Moreover, treatment with aromatase inhibitors has been associated with an increased risk of lipid profile alteration, when compared with tamoxifen.

Furthermore, most patients receiving hormone therapies experience hot flashes, decreased libido and reduced vaginal lubrication which may also compromise adherence to treatment [17]. In younger women, fertility matters have also been described, together with the higher risk of adverse obstetric outcome [18–20]. In this regard, fertility preservation strategies should be recommended at diagnosis, particularly in patients of childbearing age [21].

Moreover, it should be noted that survivors of breast cancer frequently show a burden of potentially cardiotoxic treatments (anthracycline-based chemotherapy regimens, HER2 inhibitor targeted therapy, thoracic wall radiotherapy), whose effect can be enhanced when classic risk factors, such as DM and dyslipidemia, coexist [22].

In women undergoing multimodal treatment for stage II/III breast cancer, including radiotherapy, a significantly higher risk of subsequent hypothyroidism has been reported when compared to the general population [23].

#### Surveillance recommendations for Endocrine/Metabolic disease in breast cancer survivors (Table 5)

##### Laboratory testing

A baseline evaluation of fasting blood glucose (and glycated hemoglobin), lipid profile, thyroid function and bone metabolism (calcium and vitamin D) should be recommended in most breast cancer survivors, in accordance with existing guidelines and risk factor assessment [16]. All these evaluations should be subsequently repeated based on the results of the baseline evaluation.

**Table 5:** Summary of suggested practices in breast, prostate, lung and colorectal cancer survivors

Cancer type	Risk factor	Surveillance	Additional exams
Lung cancer	All patients	Menstrual cycles (female) and symptom of hypogonadism	Gonadal function
	Steroids administration	Bone metabolism (calcium and Vitamin D)	Cardiovascular risk assessment
		DXA scan evaluation	
		Glucose metabolism (fasting blood glucose and/or glycated hemoglobin)	
	Neck irradiation	Thyroid function	Thyroid ultrasound
	Immunotherapy	Pituitary function	Pituitary MRI
		Thyroid function	Thyroid ultrasound
		Adrenal function	Cardiovascular risk assessment
		Glucose metabolism (fasting blood glucose and/or glycated hemoglobin)	
Colorectal cancer	All patients	Glucose metabolism and lipid profile	Cardiovascular risk assessment
		Menstrual cycles (female) and symptom of hypogonadism	Gonadal function
	Steroids administration	Bone metabolism (calcium and Vitamin D)	Cardiovascular risk assessment
		DXA scan evaluation	
		Glucose metabolism (fasting blood glucose and/or glycated hemoglobin)	
	Immunotherapy	Pituitary function	Pituitary MRI
		Thyroid function	Thyroid ultrasound
		Adrenal function	Cardiovascular risk assessment
		Glucose metabolism (fasting blood glucose and/or glycated hemoglobin)	
Breast cancer	Supraclavicular irradiation	Thyroid function	Thyroid ultrasound
	Immunotherapy	Pituitary function	Pituitary MRI
		Thyroid funtion	Thyroid ultrasound
		Adrenal function	Cardiovascular risk assestment
		Glucose metabolism (fasting blood glucose and/or glycated hemoglobin)	
	Premature menopause and/or Hormone therapy	Bone metabolism (calcium and Vitamin D)	
		DXA scan evaluation	
		Glucose metabolism and lipid profile	Cardiovascular risk assessment
		Sexual dysfunction	
prostate cancer	Androgen deprivation therapy	Bone metabolism (calcium and Vitamin D)	
		DXA scan evaluation	
		Glucose metabolism and lipid profile	Cardiovascular risk assessment
		Sexual dysfunction	

##### Imaging

In survivors at risk for cancer treatment-induced bone loss (CTBIL), baseline dual-energy X-ray absorptiometry (DXA) examination (ideally, combined with vertebral morphometry) should be recommended, in accordance with guidelines [24]. This evaluation should be repeated every 18–24 months to assess bone mineral density (and potentially asymptomatic vertebral fractures) at least until the end of treatment with CTBIL drugs.

Thyroid ultrasound may be considered in patients who have undergone radiation involving thyroid region [25]. In survivors with thyroid dysfunction, thyroid ultrasound may be considered, especially if there are persistent abnormalities or nodular findings. Even if thyroid ultrasound is not routinely recommended in the ESE Clinical Practice Guideline on ICI-related endocrinopathies [26], it may be considered when thyroid function alterations have been diagnosed.

In patients with specific cardiovascular risk factors, such as prior anthracycline or anti-HER2 therapy and/or chest irradiation, ongoing cardiovascular monitoring is essential. In these cases, periodic electrocardiograms and echocardiograms should be performed according to cardio-oncology guidelines [27, 28].

#### Treatment strategy for Endocrine/Metabolic disease in breast cancer survivors

Due to the increased risk of metabolic alteration in the specific context of breast cancer, all patients should receive counseling on tobacco cessation, alcohol moderation, adherence to a healthy diet, and weight-reducing physical activity when indicated [29].

Adequate intakes of calcium (1000–1200 mg per day) and vitamin D (800–2000 IU per day of Vitamin D3) should be ensured, suggesting oral supplements when levels are inadequate [30, 31].

According to AIOM guidelines, for patients with breast cancer undergoing adjuvant hormonal therapy or those in chemotherapy-induced menopause, bone resorption inhibitors should be considered from the start of hormonal therapy (primary prevention of CTIBL) and continued for the entire duration of the adjuvant hormonal therapy [16]. In this context, denosumab 60 mg every 6 months should be considered as a first line treatment for the prevention of CTIBL-related fractures [16]. However, treatment decisions should take into account individual factors such as age, baseline fracture risk (e.g., DXA and FRAX assessment), menopausal status, and concurrent comorbidities. Alternatively, the use of bisphosphonates, especially intravenous zoledronate (4–5 mg every 6–12 months), can be a valid approach [31]. Since denosumab discontinuation may lead to a rebound effect with increased fracture risk, careful planning of follow-up and monitoring is essential, although no standardized strategy currently exists to fully mitigate this risk [32, 33].

In survivors with lipid profile alteration, especially during aromatase inhibitors administration, a careful approach should be recommended, also considering more stringent targets for lipid-lowering treatment compared to those for the general population. Similarly, in patients at high cardiovascular risk (i.e., previous anthracyclines/anti-HER2 treatment and/or chest irradiation), it is crucial to rigorously manage DM and hypertension, if present [27].

### Prostate cancer survivors

#### Epidemiology and definition of disease-specific prostate cancer survivors

As one of the most common cancers in men globally accounting for 15% of overall cancers, prostate cancer predominantly affects older men, with a significant rise in incidence linked to prostate-specific antigen (PSA) screening [34]. Overall, the incidence in Europe is 144,215/4,141,360 (3.5%), and 2245/397,618 (0.6%) for age range 20–49 years. Overall, the global mortality is 397,430 deaths per year (https://gco.iarc.who.int/media/globocan/factsheets/cancers/27-prostate-fact-sheet.pdf).

According to the SEER database, it has been estimated that in 2021, 9,378 people < 50 years within the US population were living with a previous or current diagnosis of prostate cancer (SEER*Explorer: An interactive website for SEER cancer statistics [Internet]. Surveillance Research Program,* National Cancer Institute; 2024 Apr 17. [updated: 2024 Nov 5; cited 2025 Jan 23]. Available from*: https://seer.cancer.gov/statistics-network/explorer/. *Data source(s): U.S. 2021 cancer prevalence estimates are based on 2021 cancer prevalence proportions from the SEER 12 Areas (excluding the Alaska Native Tumor Registry) and 1/1/2021 U.S. population estimates based on the average of 2020 and 2021 population estimates from the U.S. Bureau of the Census)*.

Prostate cancer survivorship, which is represented by patients free of disease or with long-term treatment, has gained increasing attention due to rising prevalence, advancements in treatments, and improved survival rates [35]. While early detection has contributed to increased survival, it has also led to concerns about overdiagnosis of indolent tumors, particularly in low-risk cases [36]. The treatment timeline for prostate cancer has evolved considerably over the past decades, transitioning from radical treatments such as surgery and radiation to more personalized approaches [37].

For advanced or metastatic disease, particularly castration-resistant prostate cancer, hormonal therapies have remained central to management with emerging next-generation androgen receptor signaling inhibitors [38].

For men with advanced prostate cancer in high-income countries, survival rates have also improved significantly with a median overall survival for patients with metastatic disease now 5–7 years and continues to rise [39, 40]. Many of these men live with the side effects of long-term androgen deprivation therapy (ADT), which can impact their quality of life [41].

Enhanced management, including intensified ADT for metastatic cases, can significantly reduce complications, which is beneficial for patient outcomes and cost-effective at a societal level, as demonstrated by trials involving therapies like abiraterone and docetaxel [42, 43]. Globally, it is estimated that more equitable healthcare systems could save up to 8 million lives annually, bringing significant societal and economic benefits [44]. Additionally, systems that guide patients toward optimal care and provide support throughout treatment are expected to reduce costs while improving outcomes and enhancing the management of side effects.

#### Risk factors for Endocrine/Metabolic disease in prostate cancer

Prostate cancer and its treatments can lead to various endocrine and metabolic disorders. Radical prostatectomy is notoriously associated with sexual dysfunction and retrograde ejaculation. However, the management of prostate cancer includes several strategies. Overall, risk factors for endocrine/metabolic disease in prostate cancer include:

1. ADT: the ADT, which is currently used for patients with advanced and metastatic prostate cancer [45], can be associated with several endocrine-metabolic complications, through different mechanisms:

- Insulin resistance and DM: patients undergoing ADT show an increased risk of developing altered fasting blood glucose levels associated with insulin resistance, up to overt DM [46]. In this regard, a study showed that a Homeostatic Model Assessment for Insulin Resistance (HOMA-IR) of 17.0+/−2.8 in the ADT group compared with HOMA-IR of 6.0+/−2.8 in the non-ADT group (*p* < 0.01) [47].
- Obesity and dyslipidemia: ADTs often result in weight gain, particularly increased fat mass, and changes in lipid profiles (hypercholesterolemia, hypertriglyceridemia or mixed dyslipidemia), raising the risk of cardiovascular diseases (21933330). In another study, the prevalence of metabolic syndrome was found to be higher in the ADT group compared with the non-ADT (*p* < 0.01) and control (*p* = 0.03) groups [47].
- Osteoporosis and fractures: the prevalence of osteoporosis in men with prostate cancer on ADT accounts for 53%. Reduced testosterone levels lead to decreased bone mineral density, loss of muscle mass, increasing the risk of osteoporosis and fractures [48]. However, it was found that men with prostate cancer experience poor bone health before treatment with ADT [49]. In this regard, a study conducted by Conde et al. showed that 73.5% of men with prostate cancer not receiving ADT had osteopenia (55.9%) or osteoporosis (17.6%) of the spine and/or hip.
- Sexual dysfunction: prostate cancer and prostate cancer treatment, both including surgical procedures and pharmacological treatment, or both of them, can negatively affect sexual health [45]. Sexual health is defined as a state of physical, emotional, mental, and social well-being in relation to sexuality that encompasses sex, gender identities and roles, sexual orientation, eroticism, pleasure, intimacy, and reproduction (World Health Organization. Measuring sexual health: conceptual and practical considerations and related indicators. Geneva, Switzerland: World Health Organization; 2010).

Interestingly, Walker et al. surveyed sexual satisfaction and its predictors across several groups to explore relative impact: (1) patients following prostate cancer treatment, (2) active surveillance, (3) negative prostate biopsy result, and (4) controls. The Authors found a reduction in sexual satisfaction in the active treatment group, no changes in active surveillance or non-prostate cancer control, and improvements in the biopsy group [50].

2. Surgery and radiotherapy: a high incidence of erectile dysfunction can occur in prostate cancer survivors, ranging from 45 to 94% after radical prostatectomy and from 1 to 67% after radiotherapy (Lee et al. 2024).

#### Surveillance Recommendations for Endocrine/Metabolic Disease in Prostate Cancer (Table 5)

##### Clinical and laboratory tests

Sexual function should be routinely assessed using validated tools to monitor erectile function over time [30]. Patients should be tested for serum glucose, lipid profile, calcium, vitamin D, bone turnover markers and testosterone levels [51]. All these evaluations should be subsequently repeated based on the results of the baseline evaluation. Furthermore, cardiological evaluation should be recommended.

##### Imaging

Fracture risk in men treated with ADT or old radiation techniques should be assessed through bone mineral density (BMD) using DXA and calculation of a fracture risk assessment score [30]).

#### Treatment strategy for Endocrine/Metabolic disease in prostate cancer

An important issue in the management of prostate cancer survivors is to promote an adequate lifestyle. In this regard, ASCO guidelines [51] recommended to counsel survivors to achieve and maintain a healthy weight by limiting consumption of high-calorie foods and beverages and promoting increased physical activity (at least 150 min per week of physical activity). Furthermore, a dietary pattern high in fruits and vegetables and whole grains, together with low amounts of saturated fat consumption should be promoted [51]. ASCO guidelines also promote the intake of at least 600 IU of vitamin D per day and consumption of adequate, but not excessive, amounts of dietary sources of calcium, to prevent bone diseases. Bisphosphonate (70 mg weekly or annual intravenous zoledronic acid at a dose of 5 mg) or Denosumab should be used to treat osteoporosis.

Patients with metabolic diseases should be treated for this complication and, importantly, both duration of the treatment and the type of anti-diabetes medication had no negative effect on cancer survival [52, 53].

Finally, erectile dysfunction may be treated by a variety of approaches, including penile rehabilitation or with phosphodiesterase type 5 inhibitors (i.e. sildenafil, vardenafil, tadalafil) [45].

### Lung cancer survivors

#### Epidemiology and definition of disease-specific lung cancer survivors

Lung cancer accounts for approximately 18% of all cancer deaths globally (10.3322/caac.21660).

According to the Global Cancer Observatory (GLOBCAN 2020) [54], in 2020, there were approximately 2.2 million new cases of lung cancer globally. In the same year, the mortality was characterized by 1.8 million deaths ().

It is predominantly classified into non-small cell lung cancer (NSCLC), which comprises around 85% of cases, and small cell lung cancer (SCLC), which is more aggressive and less common. The primary risk factor for lung cancer is smoking, responsible for about 85% of cases, followed by exposure to environmental carcinogens like radon, asbestos, and air pollution (10.1093/jnci/66.6.1191).

The concept of cancer survivor in lung cancer is evolving, reflecting the diversity of patient experiences and outcomes. To date, no specific data on the global number of lung cancer survivors are available. According to the SEER database, it has been estimated that in 2021, 14,192 people within US population were living with a previous or current diagnosis of lung cancer (SEER*Explorer: An interactive website for SEER cancer statistics [Internet]. Surveillance Research Program,* National Cancer Institute; 2024 Apr 17. [updated: 2024 Nov 5; cited 2025 Jan 23]. Available from*: https://seer.cancer.gov/statistics-network/explorer/. *Data source(s): U.S. 2021 cancer prevalence estimates are based on 2021 cancer prevalence proportions from the SEER 12 Areas (excluding the Alaska Native Tumor Registry) and 1/1/2021 U.S. population estimates based on the average of 2020 and 2021 population estimates from the U.S. Bureau of the Census).* Traditionally, cancer survivorship has been associated with individuals who have completed treatment and are in remission. However, this definition is increasingly seen as insufficient in lung cancer, where a significant number of patients live with chronic or stable disease due to advanced therapies. There is no universally accepted definition of “survivor” in this context, which complicates efforts to standardize care and support services.

The lack of consensus on the definition of survivors is partly due to the varied nature of lung cancer treatment outcomes. For instance, patients who have undergone surgery for early-stage lung cancer might be considered cured or in remission, yet they still require long-term monitoring for recurrence and may experience late side effects from surgery or adjuvant therapies. Moreover, the advent of novel agents, including targeted therapies and immunotherapies, has expanded the concept of survivorship to include those receiving long-term treatment for advanced or metastatic disease. These patients, often referred to as “long-term responders” continue to receive therapy to control their cancer, similarly to chronic disease management.

This broader definition is crucial for understanding the full scope of challenges faced by lung cancer survivors. Long-term responders, while benefiting from prolonged survival due to advanced therapies, must navigate the ongoing management of treatment-related side effects. Nevertheless, recent therapeutic advancements have significantly transformed the prognosis for lung cancer patients, particularly those with NSCLC, introducing new challenges in managing long-term health complications.

#### Risk factors for Endocrine/Metabolic disease in lung cancer

Lung cancer is associated with various endocrine and metabolic disorders, often due to the cancer itself or its treatments. A study conducted on 18,372 patients with previous lung cancer, with a median follow-up period of 2.24 years from the diagnosis, showed that there is a significant increase in the incidence of head and neck (SIR 1.60, 95% CI 1.21–2.07, *p* = 0.001), bone and soft tissue (SIR 2.65, 95% CI 1.27–4.87, *p* = 0.011), and thyroid (SIR 3.85, 95% CI 2.28–6.08, *p* < 0.001) cancers in lung cancer survivors [55]. Apart from the increased risk of thyroid cancer, no increased risk of other thyroid diseases in young adult lung cancer survivors were found [56]. Indeed, hypothyroidism seems to have protective effects both on lung adenocarcinoma and squamous cell lung cancer [57].

Furthermore, different treatments for lung cancer, including radiation, targeted therapy, and immune checkpoint inhibitors (ICIs), have distinct endocrine and metabolic side effects [58].

Overall, risk factors for endocrine/metabolic disease in lung cancer include:

##### Treatment-related factors

- ICIs: while immunotherapy has been associated with durable responses and long-term survival, it also carries the risk of immune-related adverse events (irAEs), including thyroiditis, hypophysitis, adrenalitis, diabetes mellitus (DM) which affect endocrine function [26, 58].

Specifically, thyroid disorders are among the most common endocrinopathies arising from an immune damage and resulting in several clinical scenarios varying from thyroiditis, overt hypothyroidism to overt hyperthyroidism [59].

- Anaplastic lymphoma kinase (ALK) inhibitors, such as crizotinib and alectinib, have been game-changers for patients with ALK-positive NSCLC. These drugs provide targeted action against cancer cells, resulting in significant tumor regression and prolonged survival. However, long-term treatment with ALK inhibitors is associated with a range of metabolic complications, including dyslipidemia, hypogonadism, and weight gain, which are particularly concerning as they can exacerbate cardiovascular risk and reduce overall quality of life [60, 61].
- Radiation: radiation to the chest can affect the thyroid function [62]. Indeed, ionizing radiation represents the only well-established risk factor for thyroid cancer development.

Corticosteroids: Patients treated with chronic glucocorticoid use should be considered at high risk of osteoporosis [30].

##### Paraneoplastic syndromes

- Paraneoplastic syndromes represent a peculiar finding of neuroendocrine neoplasms, one of the lung cancers histotypes, including carcinoids as well as small and large cell poorly differentiated neuroendocrine carcinomas. Ectopic hormone secretion syndromes can be a marker of tumor relapse in long-term responders to advanced therapies. Lung cancers can produce hormones or hormone-like substances ectopically, like ACTH and/or CRH causing ectopic Cushing’s syndrome (ECS) and ADH leading to the syndrome of inappropriate antidiuresis (SIAD). However, this evidence occurs mainly with typical bronchial neuroendocrine tumors (NET) and SCLC [63].

Interestingly, a previous analysis of clinicopathological features and severity of hormonal syndrome from a large cohort of 110 patients with NETs and ECS collected from 17 Italian centers showed that negative predictive factors for survival were severity of hypercortisolism (*P* < 0.02), hypokalemia (*P* = 0.001), DM (*P* = 0.0146) and distant metastases (*P* < 0.001) [64].

Similarly, a report of 51 patients with ECS showed that the presence of multiple hormone secretion, including cortisol, gastrin, calcitonin, glucagon and 5-hydroxyindoleacetic acid (5-HIAA), correlated with shorter overall survival (OS), *p* = 0.012 (HR 2.4, 95% CI 1.2–4.9) [65].

Hypercalcemia can be a consequence of ectopic production of parathyroid hormone-related peptide (PTHrP) by lung cancer cells, leading to increased calcium levels in the blood; this evidence also occurs during active forms of tumors [66].

#### Surveillance recommendations for endocrine/metabolic disease in lung cancer (Table 5)

##### Laboratory tests

Patients at high risk of osteoporosis for chronic (i.e. 3–6 months) glucocorticoid use should be screened for bone health [30].

Furthermore, patients on ICIs treatment should undergo specific management for the diagnosis of related endocrinopathies [26, 58]. Thyroid function should be screened in lung-cancer patients undergoing radiation therapy as well as those treated with ICIs, even after therapy completion; morning plasma cortisol levels should be tested before starting ICI and every 4–6 weeks for the first 6 months of therapy. Patients should also be tested for fasting plasma glucose (FPG) and HbA1c levels. Testosterone, LH, FSH (in males) and FSH, LH, estradiol, menstrual cycle regularity (pre-menopausal females) should also be evaluated, according to specific diagnostic flow charts [58].

Finally, based on data in the literature, patients with suspicion for ECS should be tested for cortisol and ACTH, and calcium and water balance should also be investigated in case of specific clinical pictures.

Finally, even if no specific indications are available for patients treated with ALK inhibitors, due to the possible impact on the pituitary gonadal axis, testosterone, LH, FSH (in males) and FSH, LH, estradiol, menstrual cycles regularity (pre-menopausal females) may also be evaluated [67].

In all pre-menopausal women who received potentially gonadotoxic chemotherapy, menstrual cycles should be monitored and, in case of amenorrhea lasting more than six months the gonadal function (FSH, LH, estradiol) should be evaluated [28]. In men with symptoms suggestive for testosterone deficiency, especially if treated with potentially gonadotropic chemotherapy, gonadal function (LH, testosterone) should be evaluated.

##### Imaging

Thyroid ultrasound may be considered in patients who have undergone radiation therapy involving the neck [25] and may be considered in survivors in whom thyroid function alterations have been diagnosed. In case of suspicion of hypophysitis, a contrast-enhanced MRI of the pituitary gland should be performed. In patients with high risk of osteoporosis and fragility-related fractures, at least a DXA examination may be recommended at the end of anticancer treatments. All these evaluations should be subsequently repeated according to the results of the baseline evaluation.

#### Treatment strategy for Endocrine/Metabolic disease in lung cancer

According to ESMO Guidelines [31], DM should be treated adequately before any local treatment.

Furthermore, all patients should be counseled on the intake of calcium and vitamin D, weight-bearing exercises, and bone-healthy lifestyle and behaviors, including smoking cessation and limiting alcohol consumption [30].

Finally, chronic management of irAEs often involves corticosteroids, which can cause additional complications such as osteoporosis and hyperglycemia, further complicating the management of long-term survivors [35].

In survivors with gonadal dysfunction, hormone replacement therapy may be considered, both in males and females in pre-menopausal age. A close evaluation of benefits and harms should be done in every single patient, on the basis of general clinical conditions, symptoms and patients’ preferences. When therapy is started, preparations and treatment schedules should be selected in accordance with existing guidelines for non-oncological population.

### Colo-rectal cancer survivors

#### Epidemiology and definition of disease-specific colorectal cancer survivors

With more than 1.9 million new cases in 2020, CRC was the third most common cancer worldwide [68–70]. The total number of deaths worldwide was 576,858 (GLOBCAN 2020) [54].

The incidence and death rate from CRC have been dropping by 1 and 2% annually, respectively, in the last decade overall, despite an unexpected rapid shift to an age < 50 years old. Among 153,020 estimated new cases of CRC in the United States in 2023, 19,550 cases (13%) will be in individuals < 50 years [71].

The stage at diagnosis is the primary predictor of outcome, with a 5-year relative survival rate ranging from 91% for localized stage to 14% for distant disease. Globally, CRC 5-year relative survival rates are improving over time worldwide, increasing from 50% in the mid-1970s to 65% during 2014–2020. Long-term gains reflect early diagnosis through clinical examinations and screening, reduction of post-operative morbidity and mortality, and advances in systemic treatment for advanced disease [71].

Improved survival along with population ageing and growth has resulted in over 5.25 millions of patients with CRC, defined as CRC survivors, worldwide in 2020. About 10% of CRC survivors are living with metastatic disease, nearly half of whom were initially diagnosed in early stage [71, 72].

CRC survivors could experience long-term effects of prior cancer and current cancer therapy, including effects of colon surgery (Low Anterior Resection Syndrome, chronic diarrhea, malabsorption and, in some cases malnutrition, dehydration, electrolyte loss, and vitamin deficiency), radiotherapy, and long-term cancer treatment toxicity [73].

#### Risk factors for Endocrine/Metabolic disease in colorectal cancer survivors

The main endocrine-metabolic complications in CRC survivors result from adverse events from ICIs [26]. Immunotherapy shaped the landscape of microsatellite instable disease (MSI-H or dMMR), accounting for 5% of metastatic CRC patients. In June 2020, the FDA approved pembrolizumab for the first-line treatment of patients with MSI-H or dMMR unresectable or metastatic CRC [74]. Nivolumab plus ipilimumab combination is currently approved for previously treated patients, while representing the new upcoming standard frontline treatment according to findings from the phase 2 CheckMate-142 study and preliminary results of the phase 3 CheckMate-8HW trial.

Endocrine toxicities sequela often persists even after ICI discontinuation and medical intervention for the irAE. This is often attributed to permanent organ damage, requiring lifelong therapy replacement, especially in the case of thyroid dysfunction and primary adrenal insufficiency [75]. Some studies reported that CRC survivors showed an increased risk of DM and obesity when compared to the general population [76, 77]. A recent study also demonstrated an increased all-cause mortality in CRC survivors with DM compared with those without DM. Older age, unhealthy diet, physical inactivity, and smoke have been reported as higher risk factors for DM development [78].

Little data are available about the risk of osteoporosis in CRC patients. Despite possible bias due to the high screening intensity for BMD in cancer patients, osteoporosis and osteoporotic fractures seem to be more frequent in CRC survivors than in the general population, with a larger gender disparity [77, 79]. In addition to women, the risk seems to be higher in adult subjects, in those treated with high glucocorticoid doses or with impaired calcium and vitamin D metabolism [80].

#### Surveillance recommendations for Endocrine/Metabolic disease in colorectal cancer survivors (Table 5)

##### Laboratory tests

Patients at high risk of osteoporosis (i.e. chronic − 3 to 6 months- glucocorticoid use) should be screened for bone health [30], as this population is at increased risk for fragility fractures.

Due to the close relationship between CRC and DM and metabolic disorders, at least annual evaluation of fasting blood glucose and lipid profile may be recommended in all CRC survivors. This recommendation is supported by observational studies reporting increased incidence of metabolic syndrome in this population [76, 77].

Furthermore, patients receiving ICIs should undergo specific management for diagnosis of related endocrinopathies [26, 58]. Thyroid function should be frequently monitored in patients treated with ICIs, even after therapy completion, as persistent or delayed thyroid dysfunction is common. Morning plasma cortisol levels should be tested before starting ICI and every 4–6 weeks for the first 6 months of therapy, in line with current oncology-endocrinology consensus statements [26]. Patients should also be tested for FPG and HbA1c levels. Testosterone, LH, FSH (in males) and FSH, LH, estradiol, menstrual cycles regularity (pre-menopausal females) should also be evaluated.

In all pre-menopausal women who received potentially gonadotoxic chemotherapy, menstrual cycles should be monitored and, in case of amenorrhea lasting more than six months the gonadal function (FSH, LH, estradiol) should be evaluated. In men with symptoms suggestive for testosterone deficiency, especially if treated with potentially gonadotropic chemotherapy, gonadal function (LH, testosterone) should be evaluated.

##### Imaging

Even if thyroid ultrasound is not routinely recommended in the ESE Clinical Practice Guideline on ICI-related endocrinopathies [26], it may be considered in survivors in whom thyroid function alterations have been diagnosed.

In case of suspicion of hypophysitis, an MRI of the pituitary gland should be performed.

In patients with high risk of osteoporosis and fragility-related fractures, at least a DXA examination should be recommended at the end of anticancer treatments, and subsequently to be repeated according to the results of the baseline evaluation. This is consistent with international osteoporosis guidelines adapted to cancer survivors [30]. All surveillance strategies should be adapted based on baseline findings.

#### Treatment strategy for Endocrine/Metabolic disease in colorectal cancer survivors

All survivors should receive counselling on tobacco cessation and limiting alcohol consumption, as well as about healthy diet, weight-loss exercises when necessary [29].

Adequate intakes of calcium (1000–1200 mg per day) and vitamin D (800–2000 IU per day of Vitamin D3) should be warranted, suggesting oral supplements, when necessary, also due to malabsorption and chronic diarrhea; these recommendations align with ASCO and ESMO guidelines for bone health in survivors of adult cancers [30, 31].

Pharmacological treatments of endocrine and metabolic disorders in CRC survivors should be prescribed according to general population guidelines for each specific disorder. Due to the possible association with DM and increased mortality in CRC survivors more stringent goals for CRC survivors with DM should be considered, particularly in survivors with longer life expectancy.

In survivors with gonadal dysfunction, hormone replacement therapy may be proposed, both in males and females in pre-menopausal age. A close evaluation of benefits and harms should be done in every single patient, on the basis of general clinical conditions, symptoms and patients’ preferences. When therapy is started, preparations and treatment schedules should be selected in accordance with existing guidelines for non-cancer population.

## Conclusions

The evolving definition of “cancer survivors”, mainly in relation to those living with chronic or stable disease, has highlighted the need for a better understanding of long-term health outcomes. Survivors of breast, prostate, lung, and colorectal cancers are increasingly living longer with both the direct effects of cancer and the late endocrine and metabolic complications associated with treatment and disease progression. This paper highlights the importance of recognizing these complications, as well as identifying key risk factors that can suggest a more effective surveillance and management. A major limitation of this work is the inability to draw upon high-grade evidence. The considerable heterogeneity of available studies and the multifactorial nature of certain aspects, such as metabolic syndrome, make it difficult to formulate strong, evidence-based recommendations. Even recent rigorous efforts in this direction have been constrained by the limited number of sufficiently robust studies [81]. Further evaluations are needed to validate or adapt existing risk calculators for cancer survivors, such as FRAX for fracture risk, since intervention thresholds may differ from general population [82, 83].

Moreover, breast, colon, prostate, and lung cancer survivors are certainly highly heterogeneous in terms of clinical characteristics, comorbidities, and life expectancy. In light of these factors as well, long-term follow-up should be personalized. However, in the absence of robust, evidence-based recommendations covering all aspects of cancer survivors’ health, the practical guidance provided in this review offers clinicians a tool to address endocrine and metabolic issues in long-term follow-up, helping to improve outcomes and quality of life in both young adult and adult patients.

## Data Availability

Not applicable.
